# Cordycepin Induced MA-10 Mouse Leydig Tumor Cell Apoptosis through Caspase-9 Pathway

**DOI:** 10.1093/ecam/nen084

**Published:** 2010-10-18

**Authors:** Chun-Yi Jen, Chun-Yu Lin, Bu-Miin Huang, Sew-Fen Leu

**Affiliations:** ^1^Department of Cell Biology and Anatomy, College of Medicine, National Cheng Kung University, Tainan, Taiwan; ^2^Institute of Bioindustrial Technology, College of Human Ecology, HungKuang University, Taichung, Taiwan

## Abstract

In the present study, the apoptotic effect of cordycepin on MA-10 cells, a mouse Leydig tumor cell line, was investigated. Results demonstrated that the number of rounding-up cell increased by cordycepin (10 *μ*M to 5 mM for 24 h), and cells with plasma membrane blebbing could be observed by 100 *μ*M cordycepin. In viability test, MA-10 cell surviving rate significantly decreased as the dosage (10 *μ*M to 5 mM) and duration (3–24 h) of cordycepin treatment increased (*P* < 0.05). Cordycepin at 100 *μ*M and 1 mM for 24 h treatment induced significant DNA fragmentation (*P* < 0.05). In addition, the percentage of G1 and G2/M phase cell significantly declined by cordycepin (100 *μ*M and 1 mM) for 24 h treatment, while the percentages of subG1 phase cell increased by 100 *μ*M and/or 1 mM cordycepin in 6, 12 and 24 h treatments (*P* < 0.05), respectively, which highly suggested that cordycepin induced MA-10 cell apoptosis. In mechanism study with the treatments of caspases, c-Jun NH_2_ terminal kinase (JNK) or reactive oxygen species (ROS) inhibitors plus cordycepin for 24 h, only caspases inhibitor suppressed subG1 phase in MA-10 cells. Moreover, western blotting results showed that cordycepin induced caspase-9, -3 and -7 protein expressions, but not caspase-8, in time- and dose-dependent manners. In conclusion, cordycepin induced apoptosis in MA-10 mouse Leydig tumor cells through a caspase-9 and -3 and -7 dependent pathway.

## 1. Introduction

Cordycepin (3′deoxyadenosine), the analog of adenosine, is a constituent from the mycelia of *Cordyceps sinensis* (CS), and was considered as an active component with effects altering cytokines secretion, improving lung function, increasing energy levels and sex drive [1–3]. It has been demonstrated that cordycepin has anti-tumor effect on mouse melanoma and lung carcinoma cells and human oral cancer cells [4, 5]. Moreover, cordycepin could inhibit polyadenylate polymerase (PAP) or inactivate mRNA polyadenylation to induce tumor cell apoptosis, which is characterized by the cellular rounding-up, cytoplasmic contraction, plasma membrane blebbing, chromatin condensation, DNA fragmentation and many biochemical characteristics [6–11]. However, the molecular mechanisms regarding apoptotic signal pathways remain elusive.

The activation of cystein aspartic-specific proteases (caspases) is commonly thought to be one of the earliest points in the no-return pathway of apoptosis. Caspases are broadly categorized into upstream regulatory caspases and downstream effector caspases [12]. The upstream caspases, such as caspase-8 (death receptor pathway) and caspase-9 (mitochondria pathway), typically have a long N-terminal prodomain that facilitates interaction with and recruitment of proapoptotic proteins, including other caspases [13]. The downstream caspases, such as caspase-3, -6, and -7, typically have short prodomains that primarily cleave protein, which is important for cellular functions, and results in cell apoptosis [9, 14–16]. Moreover, some investigations have indicated that c-Jun NH_2_ terminal kinase (JNK) pathway also participates in apoptosis. JNK, a stress-activated protein kinase, is a subgroup of the MAPK superfamily, which can be activated by cell stress such as ultraviolet, TNF and interleukin-1 [17, 18]. Furthermore, reactive oxygen species (ROS), molecules possessing an odd of electrons, could induce various biological responses, including cell growth, arrest and/or cell damage [19, 20]. Excess ROS would cause damage to cellular component such as lipid membranes, protein, and DNA, leading to apoptosis [21–23].

We have previously demonstrated that CS could induce MA-10 cell apoptosis [24]. It is possible that cordycepin, the pure substance from *C. sinensis*, induce MA-10 cell death. Although some reports have demonstrated that cordycepin possesses anti-tumor action, limited reports about cordycepin-mediated apoptosis in testicular tumor cells have been illustrated. In addition, it has been demonstrated that between 1973 and 1995 the incidence of testicular cancer in the United States increases 51% (1% of total cancer cases annually), and orchidectomy is the common protocol to treat testicular and Leydig cell cancers [25, 26]. Thus, rather than orchidectomy, the exploration of cordycepin-induced cell death with the study of mechanism will be valuable to design more effective chemotherapy agents on testicular tumor cells.

## 2. Materials and Methods

### 2.1. Chemicals

Cordycepin, bovin serum albumin (BSA), Waymouth MB 752/1 medium, 3-ethyl-5-benzyl-2-methyl-4-phenylethylethynyl-6-phenyl-1,4-dihydropyridine-3,5-dicarboxylate (MRS 1191), methylthiazolecterazolium (MTT), SP600125, RNase A, propidium iodide and mercaptoethanol were purchased from Sigma Chemical (St Louis, MO, USA). Fetal bovine serum (FBS), Dulbecco's phosphate buffered saline, lyophilized trypsin-EDTA and gentamicin sulfate were purchased from Gibco (Grand Island, NY, USA). Sodium hydroxide, hydrochloric acid, sodium dodecyl sulfate (SDS), sucrose, EDTA, isopropyl alcohol, chloroform and Tween 20 were purchased from Merck (Darmstadt, Germany). Tris base and general caspase inhibitor (zVAD-fmk) were purchased from Calbiochem (San Diego, CA, USA). Acrylamide was purchased from JT Baker (Phillipsburg, NJ, USA). HEPES was purchased from Mallinckrodt Baker, Inc. (Paris, Kentucky, USA). Tissue culture grade sodium bicarbonate, sodium carbonate, sodium chloride, sodium dihydrogen phosphate and potassium chloride were purchased from Riedel-deHaen (Seelze, Germany). Donkey anti-rabbit IgG conjugated with horseradish peroxidase were purchased from Amersham International (Arlington Heights, IL, USA). Antibody against *β*-actin and the monoclonal antibody against caspase-3, -7, and -9 were purchased from Cell Signaling (Beverly, MA, USA). The polyclonal antibody against caspase-8 was purchased from Santa Cruz (Santa Crus, CA, USA).

### 2.2. Cell Culture

The MA-10 cell line was a gift from Dr Mario Ascoli (University of Iowa, Iowa City, IA, USA), and was maintained with the standard technique [27]. Cells were maintained in Waymouth medium containing 15% FBS and incubated in a humidified atmosphere containing 95% air and 5% CO_2_ at 37°C.

### 2.3. Morphology Observation

MA-10 cells (6 × 10^5^) were seeded in 6-cm Petri dish (Techno Plastic Products AG, Trasadingen, Switzerland) with 2 mL serum medium. After 70–80% confluence, cells were treated without or with 10 *μ*M, 100 *μ*M, 1 mM, 2 mM and 5 mM cordycepin for 24 h. Cell morphology was then observed and recorded under light microscopy (Olympus, CK 40). Apoptosis is characterized by the loss of cellular contact with the matrix and the appearance of plasma membrane blebbing [9].

### 2.4. DNA Fragmentation Assay

In order to investigate if cordycepin could induce cell apoptosis, DNA fragmentation was determined first. After treatment, MA-10 cells (1 × 10^6^) were lysed in a 0.6 mL cell lysis solution containing 20 mM Tris–HCl, 10 mM EDTA, pH 8.0 and 0.3% Triton X-100. DNA was extracted with 0.6 mL phenol/chloroform (1 : 1), and the mixture was centrifuged at 12 500 r.p.m. for 10 min. DNA in the aqueous phase was extracted with phenol/chloroform (1 : 1) again. The aqueous phase with DNA was mixed with isopropanol at −20°C overnight. After centrifugation, DNA pellets were washed with 70% ethanol and air-dried. DNA pellets were dissolved in TE buffer (10 mM Tris–HCl, 1 mM EDTA, pH 8.0), and RNase A (3 mg/mL) was added to remove RNA at 37°C for 30 min. DNA electrophoresis was carried out in 2% agarose gel. The gel was stained with ethidium bromide. DNA fragments were visualized under UV light and photographed.

### 2.5. MTT Cytotoxicity Test

Methylthiazoletetrazolium test was used to determine cell viability with the treatment of cordycepin [28]. MA-10 cells were seeded in 96-well plate (Techno Plastic Products AG, Trasadingen, Switzerland) containing 2 × 10^4^ cells with 100 *μ*L serum medium in each well. After 70–80% confluence, cells were treated without or with 10 *μ*M, 100 *μ*M, 1 mM, 2 mM and 5 mM cordycepin for 3, 6, 12, and 24 h, respectively. MTT was added at different time points with the final concentration of 0.5 mg/mL, and then incubated at 37°C for 4 h. The medium was removed and DMSO (50 *μ*L) was added into each well to dissolve the crystals by shaking the plate weakly for 20 min in dark. The OD values in each treatment were then determined at *λ* = 590 nm by an ELISA reader (Opsy MR, Dynex, USA).

### 2.6. Flow Cytometry Analysis

To further confirm whether cordycepin could induce cell apoptosis, the redistribution of cell cycle by flow cytometric analysis was used with propidium iodine stain [29, 30]. MA-10 cells (6 × 10^5^) were seeded in 6-cm dish with 2 mL serum medium. After 70–80% confluence, cells were treated with free medium containing various concentrations of cordycepin for 3, 6, 12, and 24 h, respectively. Cordycepin-treated cells were harvested with trypsin, washed with PBS, and mixed in 75% ethanol for at least 2 h at −20°C. After fixation, cells were washed with cold PBS and then collected by centrifugation, mixed with 100 *μ*g/mL RNase, and stained with PI (propidium iodide) solution containing 40 *μ*g/mL in PBS. The stained cells were analyzed using a fluorescence activated cell sorter (FACScan, Becton-Dickinson, Mountain View, CA, USA) at *λ* = 488 nm using Cell-Quest software (Becton-Dickinson, Mountain View, CA, USA). The DNA content distribution of normal growing cells is characterized by two peaks G1/G0 and G2/M phase. G1/G0 phase possesses normal functioning and resting state of cell cycle with most diploid DNA content, while the DNA content in G2/M phase are more than diploid. Cells in subG1 phase have least DNA content in cell cycle distribution, called hypodiploid. The hypoploid DNA contents represent the DNA fragmentation [30].

### 2.7. Immunoblotting Analysis

Cells (6 × 10^6^) were seeded in 6-cm dish. After treatment, cells were rinsed with cold PBS. Cells were then harvested by 100 *μ*L lysis buffer (50 mM Tris-base, 150 mM NaCl, 1% NP40, 0.1% SDS, 0.5% deoxychloride acid and 1 mM PMSF). Cell lysate was centrifuged at 32 000 r.p.m. for 10 min at 4°C. The pellet was collected by 10 *μ*L lysis buffer and was centrifuged again at 12 000 *g*. The supernatant, which contains total protein, was collected and stored at −20°C. Protein concentration of the cell lysates were determined by the Lowry method [31]. Cell proteins (40–60 *μ*g) were separated in 12% SDS–polyacrylamide gel, that performed at 100 V for 2.5 h using standard running buffer (24 mM Tris–HCl, 0.19 M glycine, 0.5% SDS, pH 8.3), and electrophoretically transferred to a polyvinylidene difluoride (PVDF) membrane at 400 mA for 4 h in transfer buffer (20 mM Tris–HCl, 150 mM glycine, 10% methanol, 0.01% SDS). The membranes were blocked with 5% nonfat milk, washed by TBST (20 mM Tris-base, 137 Mm NaCl, 0.1% Tween 20, pH 7.6), and subsequently incubated with primary caspase-3 or -7 antibodies at 1 : 4000 dilutions and caspase-8 or -9 antibodies at 1 : 2000 dilutions overnight at 4°C. After washing, the membrane was incubated with horseradish peroxidase-conjugated sheep anti-mouse antibody or donkey anti-rabbit antibody, and then visualized by enhanced chemiluminescence (ECL) detection kit (Amersharn-Pharmacia International PLC, UK). The optical density of each protein band was quantitated by a Quantity One (PDI, Huntington Station, NY, USA) computer-assisted image analysis system [32]. The amount of *β*-actin in each lane was also detected as a control to correct the expression of caspases proteins.

### 2.8. Statistic Analysis

Each data point in the figures represents the mean ± SEM of three separate experiments. Statistically significant differences between treatments and controls were determined by one-way ANOVA and then Least Significance Difference (LSD) comparison procedure. Statistical significance was set at *P* < .05.

## 3. Results

### 3.1. Cordycepin-Induced Morphological Change and DNA Fragmentation in MA-10 Cells

**Figure 1:**
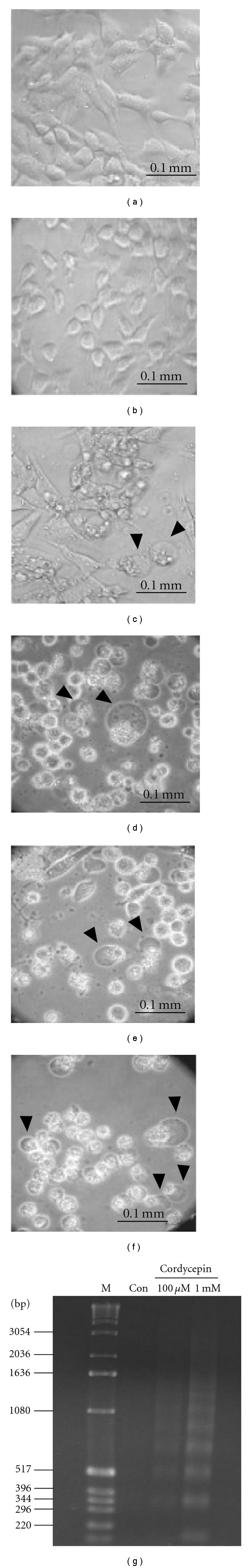
The effect of cordycepin on morphological change and DNA fragmentation in MA-10 cells. MA-10 cells were treated without (a), or with 10 *μ*M (b), 100 *μ*M (c), 1 mM (d), 2 mM (e) and 5 mM (f) cordycepin for 24 h. Morphological changes of cells were examined under light microscopy (bar: 0.1 mm; arrow heads: membrane blebbed cells). Also, gel electrophoresis of a 1 kb DNA ladder marker (lane M) or DNA isolated from MA-10 cells that were cultured for 24 h in the presence of medium alone, 100 *μ* or 1 mM cordycepin (g). DNA was visualized by ethidium bromide staining and photographed under UV illumination. Experiments were performed three times with similar results (Con = control).

MA-10 cells were treated without or with cordycepin (10 *μ*M, 100 *μ*M, 1 mM, 2 mM, and 5 mM) for 24 h, and morphological changes were observed under light microscopy. Cells without cordycepin treatment showed polygonal shape with healthy appearances, blurred outline and firm attachment, which is normal cell growth phenomenon (Figure 1(a)). Cells appeared rounded-up phenomenon but still adherent to the ground matrix with 10 *μ*M cordycepin (Figure 1(b). Cordycepin at 100 *μ*M caused more cells rounded-up, and some cells expressed plasma membrane blebbings (Figure 1(c)). More adherent cells appeared membrane blebbings with more floating cells under 1, 2, and 5 mM cordycepin treatments, and the number of attached cells reduced (Figures 1(d)–1(f)). These phenomena suggest that cordycepin might induce apoptotic cell death in MA-10 cells.

Previous results illustrated that cordycepin would cause cell death in MA-10 cells. DNA fragmentation laddering assay and flow cytometry analysis were further used to confirm apoptotic effect of cordycepin on MA-10 cells. The occurrence of DNA fragmentation is a characteristic event of cell apoptosis with genomic DNA degrading into multiples of 180–200 bp units producing a ladder feature on agarose gel electrophoresis.  Figure 1(g) notably illustrates that DNA fragmentations were observed in MA-10 cells with 24 h cordycepin treatment at 100 *μ*M and 1 mM.

### 3.2. Decreased MA-10 Cell Viability by Cordycepin

**Figure 2:**
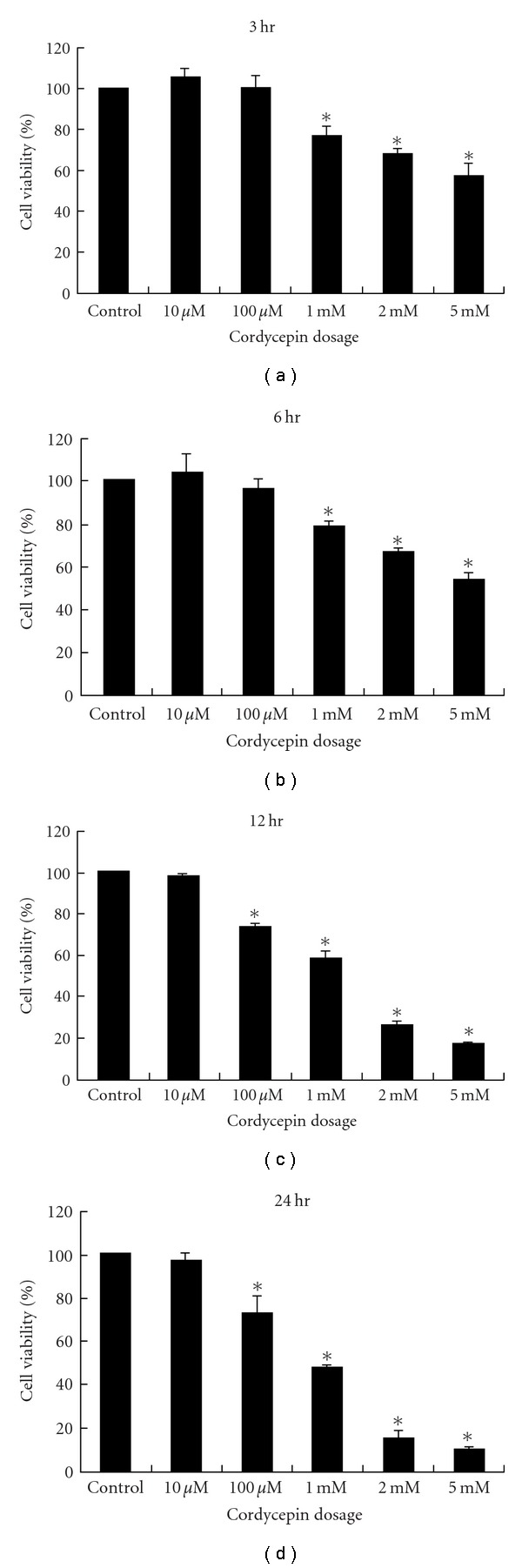
Effects of cordycepin on cell viability of MA-10 cells. Cells (5000 cells/well) were treated with different concentrations of cordycepin (0.01–5 mmol/L) for 3 h (a), 6 h (b), 12 h (c) and 24 h (d), respectively, and cell viability was quantified by MTT test. Results are expressed as percentages of cell growth relative to initial number of viable cells in controls (as 100%). Data represent the mean ± SEM of four separate experiments. *Groups differing significantly from control (*P* < .05).

The morphological changes suggested the involvement of cell death induced by cordycepin in MA-10 cells. Thus, MTT test was used to investigate the effect of cordycepin on cell viability in MA-10 cells. Cells were treated without or with various concentrations (10 *μ*M to 5 mM) of cordycepin for different time points (3, 6, 12, and 24 h). MTT tests showed that cordycepin induced death effect on MA-10 cells in a time- and dose-dependent manner (Figure 2).

Figure 2(a) illustrates that 3-h treatment of 1, 2, and 5 mM cordycepin significantly reduced cell viability to 76.8 ± 4.3%, 67.9 ± 2.5% and 57.2 ± 5.7% (*P* < .05), respectively, in MA-10 cells. After 6 h treatment with 1, 2, and 5 mM cordycepin, cell viability reduced to 78.8 ± 2.6%, 66.6 ± 2.6%, and 53.9 ± 3.4%, respectively (*P* < .05) (Figure 2(b)). Treatment with 100 *μ*M, 1, 2, and 5 mM cordycepin after 12 h caused a reduction in cell viability to 73.0 ± 2.3%, 58.1 ± 3.8%, 25.7 ± 2.3% and 16.2 ± 0.6%, respectively (*P* < 0.05) (Figure 2(c)). Moreover, treatment with 100 *μ*M, 1, 2 and 5 mM cordycepin as 24 h reduced cell viability to 73.1 ± 7.9%, 47.8 ± 1.6%, 15.0 ± 3.9% and 10.1 ± 1.2%, respectively (*P* < .05) (Figure 2(d)). The effective cordycepin concentration for 50% inhibition (EC50) on MA-10 cell viability after 24 h was 1 mM. Thus, 100 *μ*M and 1 mM were chosen for the subsequent experiments.

### 3.3. Cordycepin-Induced Cell Cycle Redistribution in MA-10 Cells

**Figure 3:**
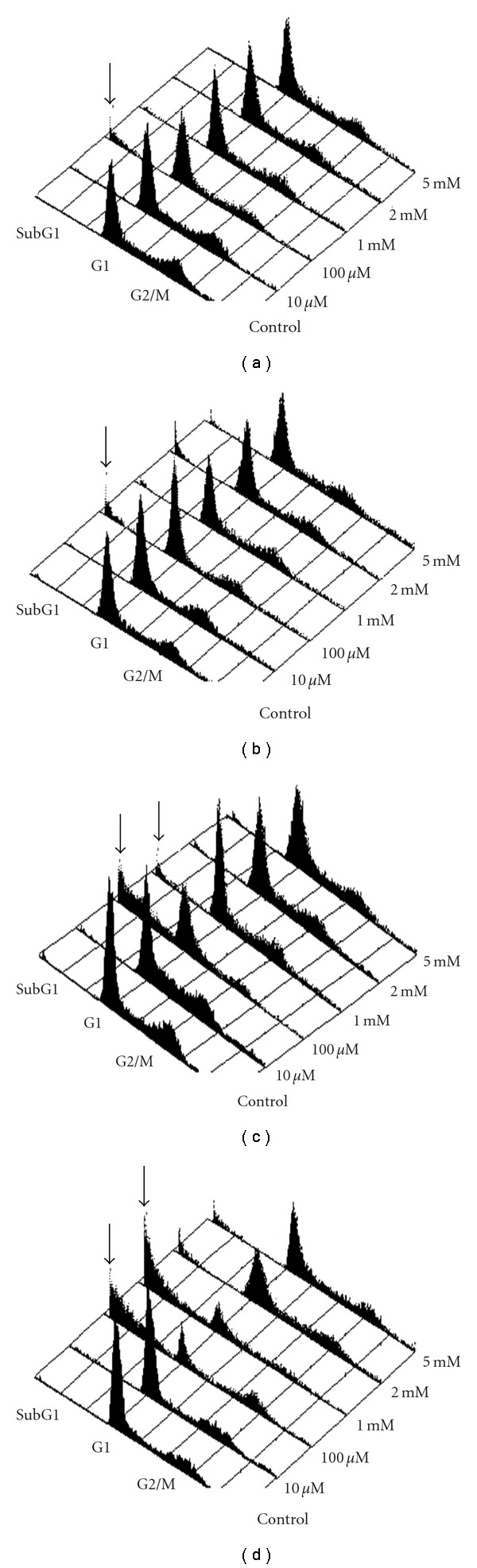
Cordycepin increased subG1 cell cycle phase in MA-10 cell line. The 3D histogram plot of flow cytometry analysis in MA-10 cells treated without or with cordycepin (10 *μ*M to 5 mM) for 3 h (a), 6 h (b), 12 h (c) and 24 h (d), respectively. At the proper time points, cells were fixed, stained with propidium iodide, and analyzed of cell cycle progression by flow cytometry as described in Section 2. SubG1 = cells with less than normal amount of DNA content; G1, cells in G1 cell cycle phase; G2/M, cells in G2/M cell cycle phase.

Moreover, in flow cytometry analysis, DNA content in each individual cell was determined.  Figure 3 illustrates the distributions of PI stained MA-10 cells treated with various concentrations of cordycepin (control, 10 *μ*M, 100 *μ*M, 1 mM, 2 mM, or 5 mM) for 3 h (Figure 3(a)), 6 h (Figure 3(b)), 12 h (Figure 3(c)) and 24 h (Figure 3(d)), respectively. The increases of subG1 phase in cell cycle distribution could be observed at 3 and 6 h treatments by 100 *μ*M cordycepin, and 12 and 24 h by 100 *μ*M and 1 mM cordycepin, respectively. Meanwhile, the decreases of cell cycle distribution could only be observed in G1 phase by cordycepin at 100 *μ*M and 1 mM, or in G2/M phase by cordycepin at 1 mM for 24 h treatment, respectively.

### 3.4. The Tendency and Analysis of Cell Cycle under Cordycepin Influence in MA-10 Cells

**Figure 4:**
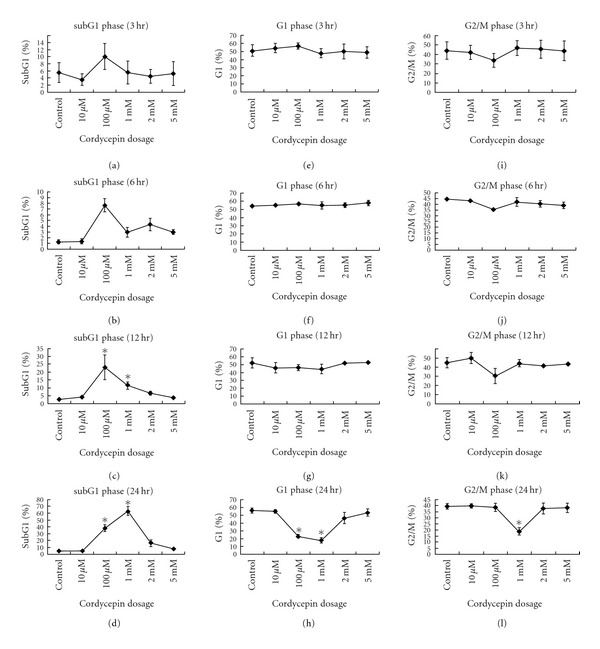
Quantification in percentage of subG1 phase cell number. Statistical analysis from three independent experiments of Figure 3 with different concentrations of cordycepin (10 *μ*M to 5 mM) regarding the change of cell cycle in percentages of subG1 phase for 3 h (a), 6 h (b), 12 h (c), and 24 h (d); in percentages of G1 phase for 3 h (e), 6 h (f), 12 h (g) and 24 h (h); and in percentages of G2/M phase for 3 h (i), 6 h (j), 12 h (k), and 24 h (l) was analyzed and illustrated. Data represent the mean ± SEM of three separate experiments. *Groups differing significantly from control (*P* < .05).

Statistical analysis from three independent experiments of Figure 3 with different concentration of cordycepin after 3, 6, 12, or 24 h treatments regarding the change of subG1, G1, and G2/M phases of cell cycle in percentages was analyzed and illustrated in Figure 4. There was no significant difference regarding the change of subG1 phase after 3 h cordycepin treatment (Figure 4(a)). However, after 6 h treatment, the subG1 phase significantly increased from 1.27% in control group to 7.64% in 100 *μ*M cordycepin treatment group (Figure 4(b)) (*P* < .05). After 12 h treatment, subG1 phase significantly increased from 2.8% in control group to 23.1 and 11.4% in 100 *μ*M and 1 mM cordycepin treatment groups, respectively (Figure 4(c) (*P* < .05). After 24 h treatment, subG1 phase significantly increased from 4.4% in control group to 38.2 and 63.1% in 100 *μ*M and 1 mM cordycepin treatment groups, respectively (Figure 4(d) (*P* < .05). In G1 phase, there were no significant differences by different dosages of cordycepin after 3, 6, and 12 h treatments (Figures 4(e)–4(g)) (*P* < .05). 100 *μ*M and 1 mM cordycepin treated MA-10 cells for 24 h did significantly decrease G1 phase from 56.3% in control group to 23.1 and 18.1% in 100 *μ*M and 1 mM cordycepin treatment groups, respectively (Figure 4(h)) (*P* < .05). Moreover, only cordycepin at 1 mM treatment for 24 h could significantly decrease G2/M phase from 39.4% in control group to 18.7% in cordycepin treatment group (Figure 4(l)) (*P* < .05).

### 3.5. The Involvement of Cordycepin on ROS, JNK and/or Caspase Pathways Related to Apoptosis in MA-10 Cells

**Figure 5:**
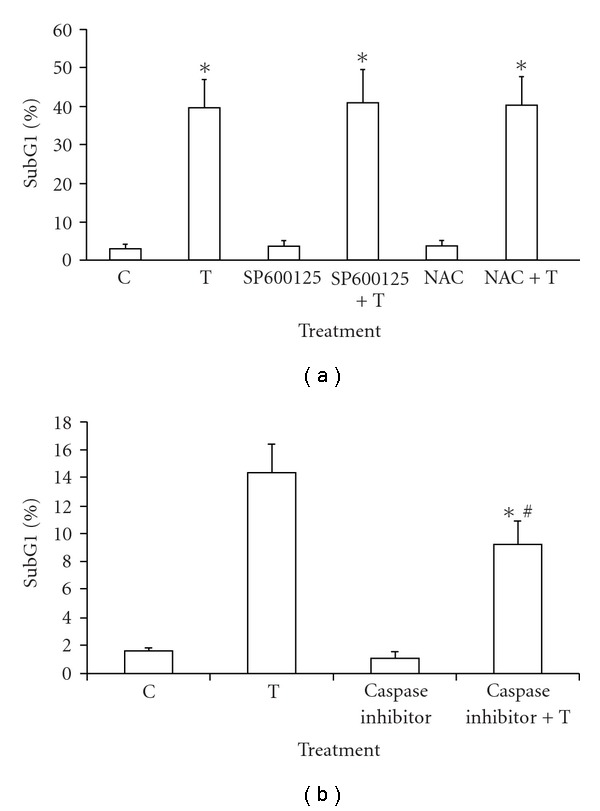
Participation of JNK, ROS and caspases in cordycepin-induced apoptosis. MA-10 cells were incubated with or without 100 *μ*M cordycepin in the presence or absence of 1 *μ*M JNK inhibitor (SP600125 which abbreviated as SP) and 1 mM ROS inhibitor (NAC) (a), or 100 *μ*M caspases inhibitor (b), respectively, for 24 h, and the change of cell cycle in percentage of subG1 phase was analyzed and illustrated. Data represent the mean ± SEM of three separate experiments. *Groups differing significantly from control (*P* < .05). ^#^Groups differing significantly from 100 *μ*M cordycepin treatment.

To investigate possible pathway that cordycepin might activate to induce MA-10 cell apoptosis, cells were co-treated with 100 *μ*M cordycepin plus 1 *μ*M JNK inhibitor (SP600125), 1 mM ROS inhibitor (NAC) or 100 *μ*M general caspase inhibitor (zVAD-fmk) for 24 h. The change of subG1 ratios determined by flow cytometry was then analyzed.  Figure 5(a) illustrates that subG1 ratio increased from 2.97% in control group to 39.82% in 100 *μ*M cordycepin treatment after 24 h (*P* < .05). JNK and ROS inhibitors could not suppress the subG1 ratio (40.76 and 40.05%, resp.) back to control levels (*P* > .05). In caspase inhibitor experiment (Figure 5(b)), the subG1 ratio increased from 1.78% in control group to 14.66% in cordycepin treatment after 24 h (*P* < .05). Interestingly, general caspase inhibitor significantly decreased subG1 ratio to 9.26% (*P* < .05). These results indicated that caspase activation/pathway played roles in cordycepin-induced apoptosis in MA-10 cells.

### 3.6. Cordycepin-Induced Differential Caspases Proteins Expression Related to Apoptosis in MA-10 Cells

**Figure 6:**
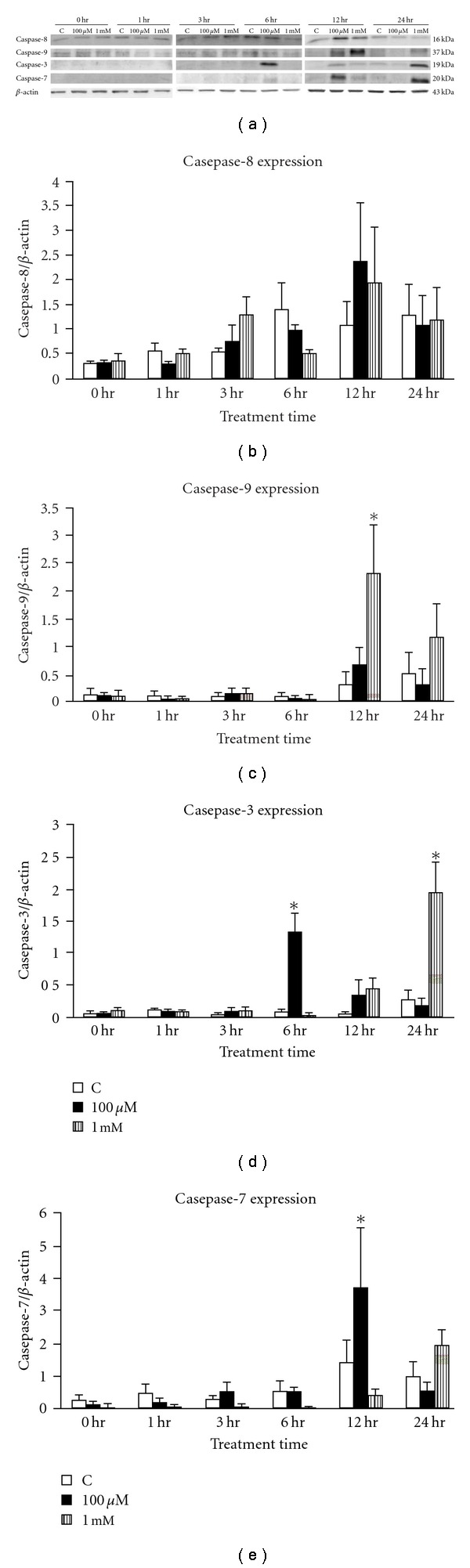
The expression of effector caspase-3 in cordycepin-induced apoptosis. Cells were treated without or with 100 *μ*M and 1 mM cordycepin for different time (0, 1, 3, 6, 12, and 24 h, resp.). Caspase-8 (16 kDa), caspase-9 (37 kDa), caspase-3 (19 kDa) and caspase-7 (20 kDa) specific bands were detected by Western blot. Immunoblot represents the observations from one single experiment repeated three times (a). The integrated optical densities (IOD) of caspase-8, -9, -3, and -7 proteins after normalization with *β*-actin (43 kDa) in each lane using PDI image system were demonstrated in (b), (c), (d), and (e), respectively. Each data point in the figure represents the mean ± SEM of three separate experiments. *Statistical difference compared to control (*P* < .05).

Previous data implied that caspase protein was involved in cordycepin-activated apoptosis in MA-10 cells. We further determined whether cordycepin could induce caspase expression.  Figure 6(a) demonstrates immunoblotting results of caspase-8, -9, -3 and -7 with cordycepin treatments (control, 100 *μ*M and 1 mM) for 0, 1, 3, 6, 12, and 24 h, respectively.  Figure 6(b) illustrates no significant differences among cordycepin treatments in each time point of caspase-8 expression (*P* > .05). However, 1 mM cordycepin (12 h) significantly induced caspase-9 expression (*P* < .05) (Figure 6(c)), 100 *μ*M cordycepin (6 h) and 1 mM cordycepin (24 h) induced caspase-3 expression (*P* < .05) (Figure 6(d)) and 100 *μ*M cordycepin (12 h) significantly induced caspase-7 expression (Figure 6(e)) (*P* < .05). These results indicate that cordycepin induced caspase-9, 3-, and -7 expressions, but not caspase-8, to activate MA-10 cell apoptosis.

## 4. Discussion

In the present study, cordycepin would induce MA-10 cell rounded-up with blebbed membrane, and reduced MA-10 cell viability with apparent DNA fragmentation. It is well known that internucleosomal DNA fragmentation displays as a biochemical hallmark during apoptosis [33]. The induction of DNA fragmentation phenomenon by cordycepin at 100 *μ*M for 24 h correlated with the increase of subG1 phase cell number of cytometry analysis, which confirms that cordycepin could induce apoptosis in MA-10 cells. Meanwhile, there were significant decreases of G1 and G2/M phase cells. Moreover, caspases inhibitor, but not JNK and ROS inhibitors, decreased subG1 ratio, implying that caspase pathway played role in cordycepin-induced apoptosis. Furthermore, cordycepin induced caspase-9, -3 and -7 expressions, but not caspase-8, to activate MA-10 cell apoptosis.

Hyperphosphorylation of PAP from p34 cyclin B kinase during mitosis will lead to the inactivation of PAP, which contributes to the reduction of mRNA and protein synthesis during the M phase of the cell cycle [34]. Also, PAP activity levels are significantly elevated at the G1/S phase of the cell cycle, along with the increased rate of mRNA polyadenylation and accumulation in the cytoplasm [35]. In the present study, treatment of MA-10 cells with cordycepin lead to the decreases of G1 and G2/M phases. It is possible that cordycepin inhibited polyadenylation, which was necessary at the G1/S phase of the cell cycle in order for all the required mRNAs to be polyadenylated, transferred to the cytoplasm and translated, in order for mitosis to take place. As a result, mitosis will not finish, and cells will arrest at the onset of apoptosis, since not all necessary proteins are translated. Indeed, the decreases of G1 and G2/M phases may imply that MA-10 cells were very sensitive to cordycepin since there was no G2/M phase arrest.

Many evidences have demonstrated that excess ROS in cell could induce cell apoptosis [22, 23, 36, 37]. An increase in ROS is also associated with the activation of redox sensitive JNK/MAPK signaling pathway, which is often involved in the transcriptional activation of genes and posttranslational modifications of proteins necessary for apoptosis [23, 38]. Hence, in the present study, both JNK and ROS inhibitors should be able to inhibit apoptotic effect. Interestingly, JNK inhibitor and ROS inhibitor could not decrease the subG1 ratio, which indicated that both JNK and ROS do not play any role in cordycepin-induced apoptosis in MA-10 cells. However, caspase inhibitor did suppress the subG1 ratio, which highly suggested that caspase pathway was involved in the cordycepin-induced apoptosis in MA-10 cells. Many investigations have illustrated that caspases play essential role in apoptosis [39]. Thus, our observations are not unprecedented.

In the present study, 100 *μ*M and 1 mM cordycepin behaved differently to induce temporal expression of caspases. In 100 *μ*M cordycepin treatment, the maximal expression of caspase-3 and -7 were at 6 and 12 h, respectively. Although there were increasing trends of caspases-8 and -9 expressions at 12 h, no significances were observed. This phenomenon illustrated that there was no caspase initiator to activate effector caspase-3 and -7. It has been shown that other initiators, such as caspase-2 and/or caspase-10 can activate effector caspases [40, 41]. It is possible that 100 *μ*M cordycepin could induce caspase-2 and/or caspase-10 to activate caspase-3 and -7 expressions for cell apoptosis. In 1 mM cordycepin treatment, caspase-9 expression (the initiator) significantly increased first at 12 h, and then caspase-3 (the effector) increased at 24 h, which illustrated a logical temporal phenomenon to induce MA-10 cell apoptosis. The possible mechanism of how different dosages of cordycepin regulating MA-10 cell apoptosis will be interesting to be further investigated.

In general, the induction of caspase-9 is due to the activation of mitochondrial apoptotic pathway, which can be activated by cytochrome *c*, ROS and JNK pathway and/or caspase-2 to induce cell apoptosis [42, 43]. In the present study, we observed that caspase-9 was activated without any involvement of ROS and JNK. It is possible that cordycepin could induce cytochrome *c*, caspase-2 or other alternative pathways to induce caspase-9 expression in MA-10 cells. This indecision will be further examined.

It has been shown that cordycepin is an analog of adenosine. The possibility of adenosine receptor involved in apoptotic effect on some types of cancer cell is also proposed [44]. Thus, it is possible that cordycepin might associate with adenosine receptor to activate apoptosis in MA-10 cells. Future works regarding the investigation on the relations to adenosine receptor in MA-10 cells will be valuable.

In conclusion, cordycepin could induce the decrease of G1 and G2/M cell numbers and the increase of subG1 cell number, followed by significant apoptotic cell death in MA-10 mouse Leydig cell line. The expressions of caspases also demonstrate that cordycepin did induce apoptosis on MA-10 mouse Leydig tumor cell line. Many Chinese herbs and its pure compounds have been demonstrated with the anti-cancer effects [45, 46]. This promising observation about the apoptotic effect of cordycepin on testicular cancer cell may be considered as a good approach in search and development of new anti-cancer drug.

## Funding

National Science Council (NSC 96-2320-B-006-059-MY3 to B.-M. Huang), Taiwan, Republic of China.
